# Development and biological activity of long-acting recombinant human interferon-α2b

**DOI:** 10.1186/s12896-020-00605-2

**Published:** 2020-03-13

**Authors:** Qian Zhang, Chao Wang, Fenlian Ma, Lihong Yao, Hanchun Gao, Luyan Zhu, Lishu Zheng

**Affiliations:** 1 NHC Key Laboratory of Medical Virology and Viral Diseases, National Institute for Viral Disease Control and Prevention, China CDC, Beijing, 100052 China; 2 Beijing Furen Ruihui Biomedical Research Institute Co.,Ltd, Beijing, 100176 China

**Keywords:** Interferon α2b, Glycosylation, Biological activity, Half-life, HBV

## Abstract

**Background:**

The type I human interferon (IFN) family consists of a group of cytokines with a multiplicity of biological activities, including antiviral, antitumor, and immunomodulatory effects. However, because the half-life of IFN is short, its clinical application is limited. Increasing the yield and biological activity of IFN while extending its half-life is currently the focus of IFN research.

**Results:**

Two novel long-acting recombinant human IFN-α2b (rhIFN-α2b) proteins were designed in which the carboxyl-terminal peptide (CTP) of the human chorionic gonadotropin β su bunit and N-linked glycosylation sequences were linked to rhIFN-α2b. They were designated IFN-1CTPON (fused at the C-terminus of rhIFN-α2b) and IFN-2CTPON (fused at both the C-terminus and N-terminus of rhIFN-α2b). Monoclonal CHO cell strains stably and efficiently expressing the IFNs were successfully selected with methotrexate (MTX), and the highest expression levels were 1468 mg/l and 1196 mg/l for IFN-1CTPON and IFN-2CTPON, respectively. The proteins were purified with affinity chromatography and molecular sieve chromatography. IFN-1CTPON and IFN-2CTPON showed antiviral and antiproliferative activities in vitro. Notably, the half-life of IFN-1CTPON and IFN-2CTPON in vivo were three-fold and two-fold longer than that of commercially available rhIFN-α2b.

**Conclusions:**

CHO cell strains stably expressing long-acting rhIFN-α2b were screened. The purified IFN-CTPON protein has biological activity and an extended half-life, and therefore potential applications.

## Background

Since the interferons (IFNs) were discovered in 1957 [1], they have been described according to their antiviral properties, and have become therapeutic options in the control of viral infections. The IFNs can be divided into three types (types I, II, and III) based on their different receptors, and are key components of the natural human immune system. The type I IFNs include 13 subtypes, among which IFN-α and IFN-β are the most extensively studied. They are mainly produced by human leukocytes and have strong antiviral properties. There is only one type II IFN, IFN-γ, which is produced by T cells [2], and its immunomodulatory effect is stronger than its antiviral activity. The type III IFNs are IFN-λ1, IFN-λ2, and IFN-λ3, as reported by Kotenko [3] and Sheppard [4]. The type III IFNs have antiviral and immunomodulatory functions, similar to those of the type I IFNs, but their effects are different because they use different receptors. The type I IFNs were one of the earliest subtypes identified and are the IFNs most widely used in the clinical context. For example, IFN-α and IFN-β are currently widely used to treat hepatitis B (HBV), hepatitis C (HCV), and hepatitis E (HEV) infections, and are also effective against chronic hepatitis δ infections [5]. In addition to treating chronic HBV and HCV infections, IFN-α2b can also be used to treat *Human immunodeficiency virus* and certain types of cancer, such as malignant melanoma, renal cell carcinoma, and chronic myeloid leukemia [6–9].

Because of the short half-life of IFNs, their clinical application are limited [10], and only high and frequent doses can achieve the therapeutic effects required. The use of these drugs has also led to frequent adverse reactions. Therefore, the modification of recombinant human IFNs (rhIFNs) to optimize their pharmacokinetic properties has become a key way to maximize their efficacy while reducing their adverse effects. At present, the covalent binding of polyethylene glycol (PEG) to the protein is the most commonly used method to prolong the half-life of IFN in serum [11, 12]. However, patient sensitivity to PEG may still compromise the effectiveness and safety of the drug therapy [13].

Fusion protein technology is another technique that has been used to increase the half-life of rhIFNs. For example, albinterferon-α2b, with a molecular weight of 85.7 kDa, is a recombinant protein in which IFN-α2b is fused to human albumin, this has extended the interval at which it is administered subcutaneously to 2 weeks. A comparison of two groups treated with albinterferon-α2b or PEG–IFN-α2a showed that their efficacy was similar in all the patients, who were chronically infected with different genotypes of HCV [14, 15]. However, more adverse effects and lung complications were observed in the patients treated with albinterferon-α2b, so the drug did not achieve the desired results [16].

In the present study, the structure of CTP, the terminal peptide of the β subunit of human chorionic gonadotropin (hCG) and four N-linked glycosylation sequences were conjugated to the C-terminus or both termini of IFN-α2b. CTP contains four O-glycosylation sequences, and the binding of oligosaccharides derived from CTP can extend the half-life of the fused protein [17–19], Studies have shown interferon fused with CTP can extend the half-life significantly [19]. N-linked glycosylation sequences have also been used to generate a long-acting rhIFN-α2b [20]. Therefore, we anticipated that the fusion of CTP with N-glycosylation sequences (CTPON) would significantly prolong the half-life of IFN-α2b, as the previous study on IFN-λ [21].

In addition to enhancing the half-life of IFN-α2b, the second objective of this study was to identify cell strains that strongly express recombinant IFNs. Almost all the classic expression systems, including *Escherichia coli*, yeast, mammalian cells, and several plant species, have been used for the production of rhIFNα2b [22, 23]. CHO cell strains, which strongly express the IFNs, were screened with methotrexate (MTX) and dot-blotting in a serum-free CHO suspension culture, and the biological activity and pharmacokinetic parameters of the purified IFNs were evaluated.

## Results

### Construction of expression vectors

**Fig. 1:**
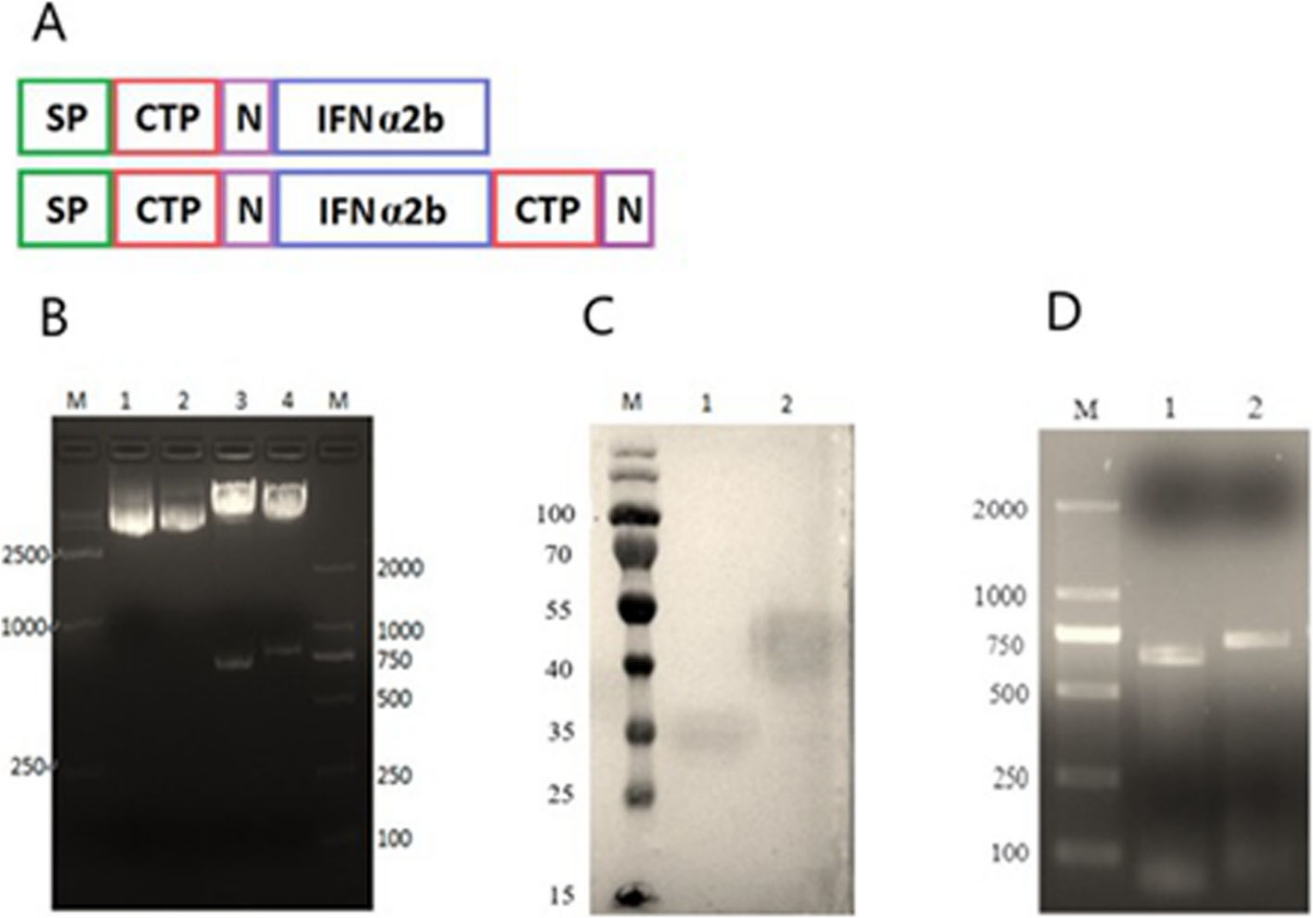
Construction and detection of expression vectors, validation of target proteins, and assessment of cell strain stability. **a** Schematic representation of expression vectors IFN-1CTPON and IFN-2CTPON. SP, Signal peptide; N, N-glycosylation sequence. **b** Restriction mapping confirmed the construction of the expression vectors. M, DNA size marker; lane 1, IFN-1CTPON DNA fragment inserted into the digested pEE12.4 plasmid; lane 2, IFN-2CTPON DNA fragment inserted into the digested pEE12.4 plasmid; lane 3, expression vector pEE12.4-IFN-1CTPON doubly digested with *Hin*dIII and *Eco*RI; lane 4, expression vector pEE12.4-IFN-2CTPON doubly digested with *Hin*dIII and *Eco*RI. **c** Western blot of culture supernatants containing IFN-CTPON. M, protein molecular size marker; lane 1, culture supernatant containing IFN-1CTPON; lane 2, culture supernatant containing IFN-2CTPON. d Monoclonal CHO cell stability verified with PCR. M, DNA size marker; lane 1, IFN-1CTPON (642 bp); lane 2, IFN-2CTPON (713 bp)

Two chimeric constructs expressing rhIFN-α2b were designed. In IFN-1CTPON, CTPON was conjugated to the C-terminus of rhIFN-α2b, whereas in IFN-2CTPON, CTPON was fused to both the C- and N-termini of rhIFN-α2b (Fig. 1a). These two coding sequences were inserted separately into the expression vector pEE12.4-dhfr, to generate the recombinant plasmids pEE12.4-IFN-1CTPON and pEE12.4-IFN-2CTPON, respectively, which were confirmed with restriction enzymes *Eco*RI and *Hin*dIII, which produced DNA fragments consistent with the expected sizes (Fig. 1b).

### High-yield-IFN clone pool and monoclonal CHO cell strain selection

After six rounds of selection in which the MTX concentration was gradually increased, the expression levels of IFN in the two high-yield CHO clone pools (IFN-1CTPON, pool 318; IFN-2CTPON, pool 308) were similar, at about 50 mg/L, when measured with an enzyme-linked immunosorbent assay (ELISA). After four rounds of selection and expansion, the monoclonal cell strains expressing IFN-1CTPON and IFN-2CTPON at the highest levels were isolated. Other factors, such as the cell doubling time and cell agglomeration, were also considered. Finally, 9 and 11 cell strains were obtained for IFN-1CTPON and IFN-2CTPON, respectively, and their highest expression yields were 1468 mg/l and 1196 mg/l, respectively.

### Identification of protein expression and gene integration

A western blotting (WB) analysis indicated that the molecular weights of IFN-1CTPON and IFN-2CTPON were larger than predicted (22 kDa and 25 kDa, respectively), and that the bands were somewhat diffuse (Fig. 1c). A PCR analysis of the genomic DNA of the CHO cells produced amplification products that were consistent with expectations (642 bp and 713 bp, respectively) after 10 passages of cell lines, and confirmed that the gene encoding IFN-CTPON had been stably integrated into the CHO cells (Fig. 1d). In addition, the protein expression remained stable above 1000 mg/l.

### Purification and N-glycosylation identification of IFN-1CTPON and IFN-2CTPON

**Fig. 2:**
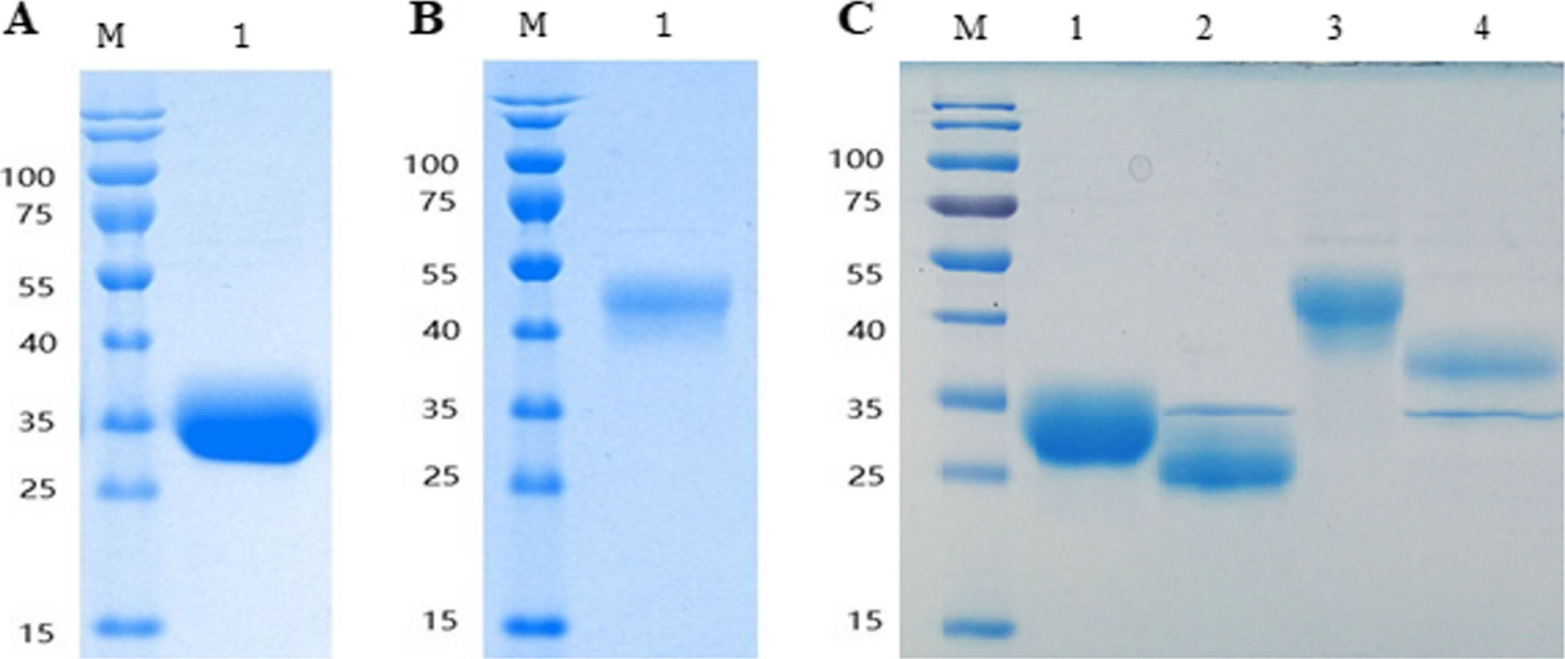
SDS-PAGE of purified IFN-1CTPON, IFN-2CTPON and identification of N-glycosylation. **a** SDS-PAGE of purified IFN-1CTPON. M, protein molecular size marker; lane 1, purified IFN-1CTPON. **b** SDS-PAGE of purified IFN-2CTPON. M, protein molecular size marker; lane 1, purified IFN-2CTPON. **c** SDS-PAGE of purified IFN-1CTPON and IFN-2CTPON digested with PNGase F. M, protein molecular size marker; lane 1, IFN-1CTPON; lane 2, IFN-1CTPON digested with PNGase F; lane 3, IFN-2CTPON; lane 4, IFN-2CTPON digested with PNGase F

**Fig. 3:**
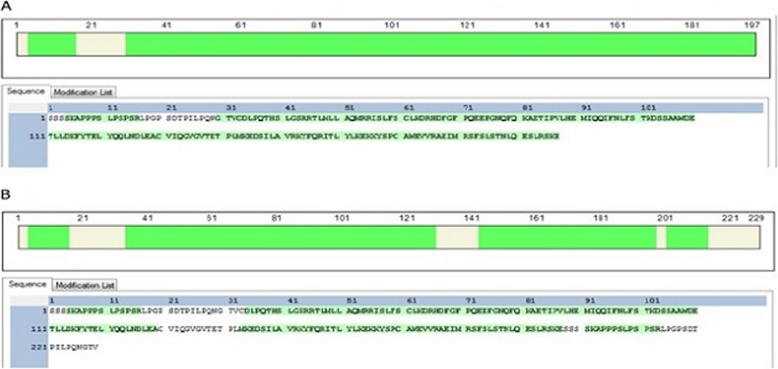
Amino acid sequence analysis of purified IFN-1CTPON and IFN-2CTPON. **a** Coverage of the amino acids of IFN-1CTPON analyzed with liquid chromatography-double pressure linear ion trap high resolution mass spectrometry. **b** Coverage of the amino acids of IFN-2CTPON analyzed as described above. Note: Green area represents the amino acid sequence obtained by Mass Spectrometer, while the white area represents the undetermined amino acid sequence

Both IFN-1CTPON and IFN-2CTPON were purified with a two-step chromatographic purification procedure, using Blue Beads 6FF affinity chromatography and Superdex 200 molecular sieve chromatography. The purity of the IFN-1CTPON and IFN-2CTPON proteins was greater than 99 and 97%, respectively, when evaluated with the ImageJ software (Fig. 2a, b). Additionally, the molecular weight of both IFN-1CTPON and IFN-2CTPON were decreased significantly after digestion with *Peptide* N Glycosidase *F (PNGase F)* (Fig. 2c). The coverages of amino acid sequence of IFN-1CTPON and IFN-2CTPON were 91.88 and 77.29%, respectively, which confirmed that the target proteins were accurately expressed (Fig. 3a, b).

### Antiviral activity

The specific antiviral activities of IFN-1CTPON and IFN-2CTPON were determined with the WISH–VSV system. The antiviral activities were calculated with reference to an IFN standard. The accurate antiviral activities of IFN-1CTPON and IFN-2CTPON were 8.22 × 10^6^ IU/mg and 3.12 × 10^6^ IU/mg, conserved 15.5 and 5.9%, respectively, compared with rhIFN-α2b (5.29 × 10^7^ IU/mg).

### Antiproliferation activity

**Fig. 4:**
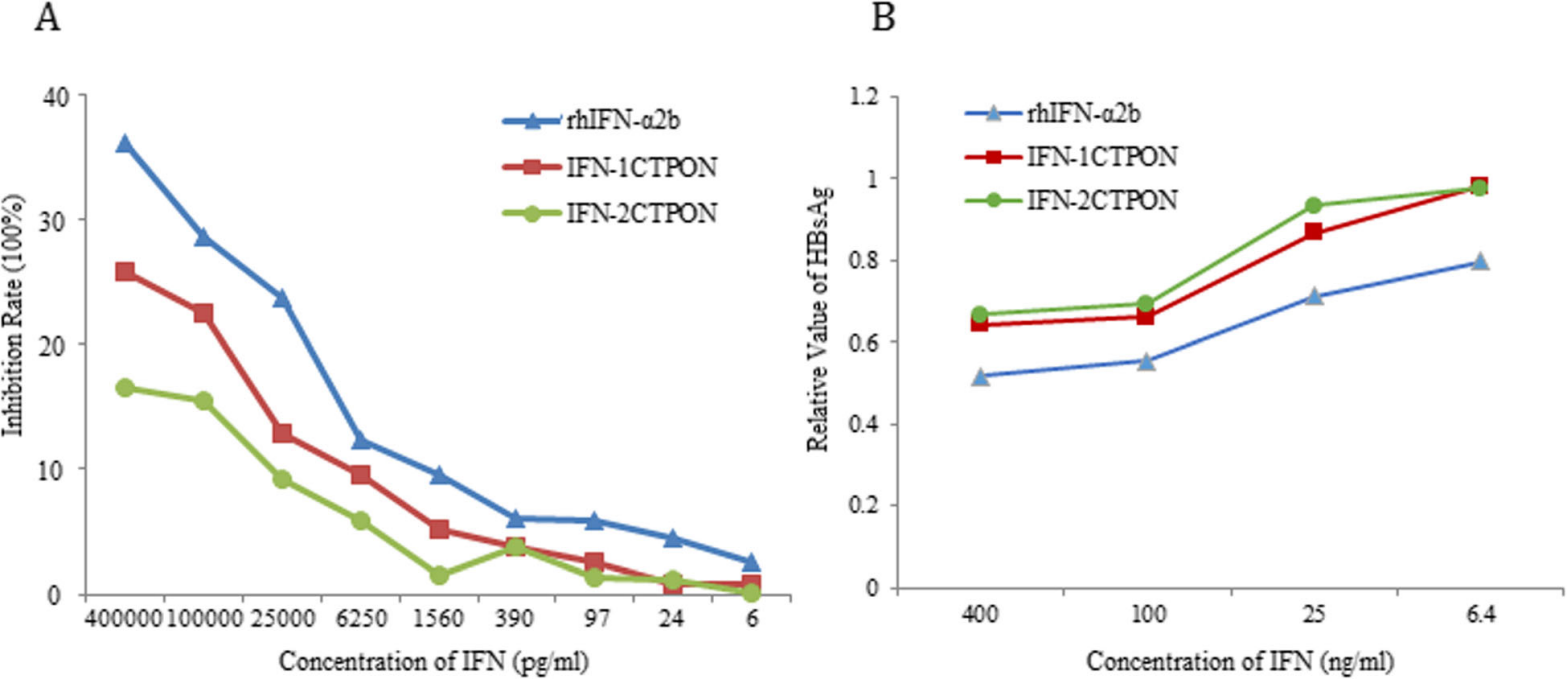
Comparison of the biological activities of IFN-1CTPON, IFN-2CTPON, and the rhIFN-α2b standard. **a** Antiproliferation activity assay of IFN-1CTPON, IFN-2 CTPON and the rhIFN-α2b standard. **b** Anti-HBV activity assay of IFN-1CTPON, IFN-2CTPON and the rhIFN-α2b standard. Triangles, IFN-α2b; squares, IFN-1CTPON; circles, IFN-2CTPON

A proliferation assay revealed that rhIFN-α2b, IFN-1CTPON, and IFN-2CTPON had dose-dependent growth suppression activities against HeLa cells. When the three kinds of IFNs were applied to HeLa cells at a concentration of 400 ng/ml, the cellular proliferation activities were inhibited by 36.18, 25.77, and 16.53%, respectively. Thus, the inhibitory effect of rhIFN-α2b was higher than those of IFN-1CTPON and IFN-2CTPON (Fig. 4a).

### Anti HBV activity

The secretion of the surface antigen of HBV (HBsAg) was detected with an ELISA kit. HBsAg production was reduced to 63.1% or 64.4% by IFN-1CTPON or IFN-2CTPON, respectively, at a concentration 400 ng/ml. However, rhIFN-α2b showed the strongest inhibitory effect, reducing antigen secretion to 50% (Fig. 4b).

### Pharmacokinetic properties

The pharmacokinetic profiles of the subcutaneously administered IFN-1CTPON and IFN-2CTPON in mice were calculated with the WinNonLin pharmacokinetic model. The half-life of IFN-2CTPON was 3.45 h, which was longer than that of IFN-1CTPON (2.62 h) or rhIFN-α2b (1.34 h). The maximum concentration (C_max_) values of IFN-1CTPON and rhIFN-α2b were basically the same, whereas the C_max_ of IFN-2CTPON was almost twice that of the other two proteins (Table 1).

**Table 1:** Pharmacokinetic parameters in mice calculated with WinNonLin

Treatment	Dose (μg/kg)	T_1/2_ (h)	T_max_ (h)	C_max_ (ng/ml)	AUC (ng h/ml)
IFN-1CTPON	50	2.62 ± 0.34	1.63 ± 0.52	13.91 ± 4.47	59.21 ± 21.01
IFN-2CTPON	50	3.45 ± 0.87	2 ± 0	23.9 ± 5.6	254.67 ± 69.58
rhIFN-α2b	50	1.34 ± 0.48	0.69 ± 0.26	14.03 ± 2.68	39.87 ± 11.9

## Discussion

IFN is a natural protein that acts as the first line of defense against many pathogens in the animal body [24]. rhIFNs have been on the market for more than 20 years, and remain an ongoing research hotspot. The increasing incidence of certain cancers and viral hepatitis has increased the demand for rhIFN-α2b, suggesting that rhIFN-α2b must be optimized in all the expression systems used for its production [22]. Although many mammalian expression systems can undergo post-transcriptional modification, Chinese hamster ovary (CHO) expression system was considered as the preferred production platform for the expression of proteins that require posttranslational modification, because it has the following advantages: the expressed proteins are closest to natural proteins in terms of molecular structure, physicochemical properties and biological functions; CHO cells can grow at high density in suspension culture under serum-free culture conditions; protein products can be secreted extracellularly, facilitating downstream protein purification [25]. For the above reasons, we chose the CHO system to express IFN. Through MTX screening, IFN-1CTPON and IFN-2CTPON were highly expressed, both of which were more than 1100 mg/l, and significantly higher than that of pichia pastoris-based expression system [22].

The target bands for IFN-1CTPON and IFN-2CTPON were much larger than the sizes expected according to their amino acid sequences, and the IFN-CTPON protein band has clearly diffuse. These phenomena were attributable to the glycosylation of the proteins in the CHO expression system and the heterogeneity of glycosylation, respectively, as reported previously by Ceaglio et al. [19, 20]. Moreover, the molecular weight of IFN-CTPON proteins were decreased after digestion with PNGase F, which proved that N glycosylation was successful in this experiment. As we mentioned in the introduction, although IFN-α is important in the defense against viral infections and modulates the antiviral immune response, its use as a protein therapeutic agent is limited by its short half-life in serum [26]. Various constructs, such as PEGylated IFN, have been developed to improve its serum half-life and reduce its renal excretion [27]. However, human sensitivity to PEG can have considerable clinical side effects [13]. Therefore, the purpose of this study was to develop a novel long-acting IFN and at the same time to maximize the yield in the expression system. The addition of CTP is a technique used to extend the half-lives of proteins, discovered by Irving Boime of the University of Washington, and it has since been confirmed that the CTP sequence is associated with the long half-life of hCG [17–19]. It has also been reported that four N-glycosylation sites can extend the half-life of IFN [20]. Therefore, when fusing CTP to IFN, we added an N-glycosylation site to the end of CTP to produce a better long-term effect. This method has been used successfully to extend the half-life of human IFN-λ1 in our laboratory [21].

In this study, IFN-1CTPON and IFN-2CTPON demonstrated antiviral, antiproliferative, and anti-HBV effects in vitro. However, the bioactivities of IFN-1CTPON and IFN-2CTPON were lower than that of commercially available rhIFN-α2b. Similar activity reductions have been confirmed in other report [19], probably because the fusion of CTPON to IFN increased its molecular weight, and the glycosylation of the fusion protein may have changed its three-dimensional structure, or they could act by producing steric hindrance to interact with the IFN receptor, which may largely explain the differences in the biological activities of the rhIFNs. Despite the lower in vitro biological activity, the in vivo biological activity could be increased in terms of the pharmacokinetic improvement. In the present study, pharmacokinetic experiments showed that the serum half-life of IFN-1CTPON and IFN-2CTPON were significantly prolonged compared with that of rhIFN-α2b (about two and three times that of rhIFN, respectively). Studies have shown that 5 N glycosylation-modified interferon pharmacokinetic activity is not significantly different from 4 N-glycosylation-modified interferon. In addition, excessive glycosylation may lead to oligosaccharide attachment saturation for a specific molecule, which indicates that excessive addition of glycosylation of the protein is not always necessarily beneficial [20]. Therefore, in order to extend the half-life of IFN and improve its in vivo biological activity, it is necessary to optimize the amounts of CTP and N glycosylation added and to select the optimal fusion site (N- or C-terminus of the protein). C_max_ of IFN-1CTPON was basically the same as that of rhIFN-α2b, whereas C_max_ of IFN-2CTPON was almost twice that of rhIFN-α2b. It is noteworthy that the area under the curve (AUC) for IFN-2CTPON was significantly greater than that for IFN-1CTPON or rhIFN-α2b, indicating that the bioavailability of IFN-2CTPON was higher than those of the other proteins. Based on its biological activity and pharmacokinetic parameters, IFN-2CTPON has potential utility in clinical applications.

## Conclusions

IFN-1CTPON and IFN-2CTPON were constructed by linking CTP to the termini of IFN-α2b, and a CHO cell strain stably and efficiently expressing IFN was selected with MTX. The biological activities of the expressed rhIFNs and longevity of their effects were confirmed. Therefore, further study of the effects of the numbers and locations of CTPON sequences on the biological activity and half-life of IFN will extend the therapeutic applications of this novel long-acting rhIFN-α2b.

## Methods

### Viral strains, cell lines, vectors, and reagents

Vesicular stomatitis virus (VSV), WISH cells, HeLa cells, and HepG2.2.15 cells used in the present study have been described previously [28]. CHO DG44 cells and the vector pEE12.4-dhfr were obtained from Life Technologies (Waltham, USA). rhIFN-α2b was obtained from Yuan-Ce Corporation (Beijing, China). The Human IFN-α (subtype 2) ELISA development kit (3423-1H-6) was obtained from MabTech.

### Expression vector construction and CHO cell transformation

The 28-amino-acid (SSSSKAPPPSLPSPSRLPGPSDTPILPQ) CTP sequence and the C-terminal-linked N-glycosylation sequence (NGTV) were optimized and synthesized by Invitrogen (Beijing, China). The fusion construct was designated ‘CTPON’. CTPON was fused to IFN-α2b at the C-terminus or at both termini, and the constructs were designated ‘IFN-1CTPON’ and ‘IFN-2CTPON’, respectively. The sequence of IFN-1CTPON or IFN-2CTPON was inserted into plasmid pEE12.4-dhfr at restriction enzyme sites (*Hin*dIII and *Eco*RI) at both ends of the expression vector. The resulting recombinant plasmids were confirmed with restriction enzyme digestion and DNA sequencing. The recombinant plasmids pEE12.4-IFN-1CTPON and pEE12.4-IFN-2CTPON were adjusted to a concentration of 40 μg, and CHO DG44 cells at a cell density 1 × 10^7^ cells/ml were transformed with 800 μl of plasmid. The conditions for electrotransformation in a 4 mm cuvette were: voltage 290 V; one square wave; 20 ms. After pulsing, the mixture was incubated in CD01 medium with 3% fetal bovine serum for 48 h.

### High-yield clone pool screening

At 48 h after electrotransformation, the CHO DG44 cells were diluted and plated in 96-well plates with the initial screening medium (CD01 + 50 μM MTX), and a dot blot analysis was performed. The primary antibody used was a monoclonal anti-hCG antibody (diluted 1:500) and the secondary antibody was a horseradish peroxidase (HRP)-conjugated goat polyclonal antibody directed against Rb IgG (diluted 1:2000). The cells showing highly intense hybridization were selected and transferred to 48-well plates, 24-well plates, six-well plates, culture dishes, and shaker flasks with 100, 200, 400, 800 and 1200 nM MTX, respectively. The IFN expression in the clone pools was detected with an ELISA kit.

### Screening for strongly IFN-expressing monoclonal CHO cell strains

Monoclonal cell strains were selected from the cloning pools. The cells were seeded into a 96-well plate with the limiting dilution method, and the culture wells with only one cell were selected. The expression of IFN in each well was detected with dot blotting; the cell doubling time, cell agglomeration, and the density of living cells were also taken into account. The cell strains most strongly expressing IFN-1CTPON or IFN-2CTPON were identified after four rounds of selection and expansion. The expression of the IFNs was quantified with an ELISA kit as described above.

### Identification of protein expression and gene integration

The IFN-1CTPON and IFN-2CTPON proteins in the cell culture medium were detected with WB. The primary antibody used was a mouse monoclonal anti-human CTP antibody and the secondary antibody was an HRP-conjugated goat anti-mouse IgG. The DNA genomes of the monoclonal CHO cells strongly expressing IFN were extracted with the QIAamp DNA Mini Kit (Qiagen, Germany). The primers used for the PCR amplification of IFN-1CTPON and IFN-2CTPON were: for IFN-1CTPON, F1 5′-ATGGGCTGGTCCTGCATCATCCTGTT-3′; R1 5′- CTCTTTGCTTCTCAGGCTCTCTTGCAG-3′; for IFN-2CTPON, F2 5′- GGTCCTGCATCATCCTGTTT-3′; R2 5′-CAGAATGGGTGTGTCGGAAG-3′. After 10 generations of the high-expression cell lines, the amplified gene fragments were recovered and sequenced, and the resulting sequences were aligned with the designed sequence. Meanwhile, the IFN-1CTPON and IFN-2CTPON proteins were quantified.

### Purification of IFNs

The fermentation broth expressing IFN was centrifuged at 10,000 rpm for 30 min at 4 °C, and the precipitate was discarded. The supernatant was then filtered through a 0.45 μm filter and purified with Blue Beads 6FF (Smart-Life Sciences Biotechnology Co. Ltd). After the column was equilibrated with binding buffer (50 mM Na_2_HPO_4_, 50 mM sodium citrate, pH 7.0), the sample was loaded onto the column and the unbound protein was washed continuously with binding buffer. The protein of interest was washed with elution buffer (50 mM Na_2_HPO_4_, 50 mM sodium citrate, 1 M NaCl, pH 7.0). The eluted proteins were concentrated to 1 ml with ultrafiltration and directly loaded onto S-100 gel (GE Healthcare) and eluted with phosphate-buffered saline (0.5 M NaCl) at a flow rate of 1 ml/min. The relevant eluted peak was collected and the target protein detected with WB.

### Determination of protein purity, protein concentrations and identification of N-glycosylation

The purified IFN-1CTPON and IFN-2CTPON proteins were separated electrophoretically with 12% denaturing SDS-PAGE and stained with Coomassie Brilliant Blue. Protein purity was calculated with the ImageJ software [22]. IFN-1CTPON and IFN-2CTPON were further concentrated with a centrifugal filter (Millipore), and the IFN contents were determined with an ELISA kit, as described above. The purified proteins were digested by PNGase F (BioLabs) according to the manufacturer’s protocol.

### Amino acid coverage analysis

The purified samples were digested with trypsin and chymotrypsin, and the amino acid sequences were analyzed with a Velos Pro Dual-Pressure Linear Ion Trap Mass Spectrometer (LTQ Orbitrap Velos Pro; Thermo Fisher Scientific).

### Antiviral assay in WISH cells

The traditional WISH/VSV system was used to evaluate the antiviral activities of the IFNs. WISH cells cultured in Dulbecco’s modified Eagle’s medium (DMEM) supplemented with 10% fetal bovine serum (FBS) were added to 96-well plates at a density of 10^4^ cells/well and left overnight. IFN-1CTPON, IFN-2CTPON, or 1000 U/ml rhIFN-α2b (as the standard) were serially diluted (4^0^–4^9^-fold) and incubated with the WISH cells for 24 h. They were then challenged with 100 tissue culture infective doses (TCID_50_) of VSV. The cytopathic effect was observed 16–24 h later. When all the control cells were dead, the supernatant was discarded from the cell culture plates and 50 μl of 0.1% crystal violet was added. After 30 min, the plates were washed with water and treated with decolorizing solution. The absorbance detected at 570 nm was used to calculate the antiviral activity of the IFNs. The IFN standard was defined as having one unit of activity when 50% of the cells were dead.

### Antiproliferation assay

Antiproliferation activity was measured with the Cell Titer 96 AQueous One Solution Cell Proliferation Assay kit (Promega, Madison, USA). HeLa cells were incubated with DMEM containing 10% FBS were seeded in 96-well plates at a concentration of 5 × 10^4^ cells/well. After 6 h, the culture medium was discarded and fresh medium supplemented with IFN-1CTPON, IFN-2CTPON, or rhIFN-α2b serially diluted 4^0^–4^9^-fold from an initial concentration of 400 ng/ml was added. After growth for 72 h at 37 °C, 20 μl of One Solution Reagent was added to the cells in each well and the plate was equilibrated for up to 4 h. The absorbance at 490 nm was recorded with a microplate reader.

### Anti-HBV activity

HepG2.2.15 cells cultured in DMEM supplemented with 10% FBS were seeded in 96-well plates until they reached 40% confluence. IFN-1CTPON, IFN-2CTPON, or rhIFN-α2b (as the standard) serially diluted 4^0^–4^9^-fold from 400 ng/ml was then added and the cells incubated for 72 h, with four parallel wells for each IFN dilution. The HBsAg concentration in the supernatant from each well was measured with a commercially available ELISA kit (Wantai Pharmaceutical Co., China).

### Animals

Male BALB/c mice (weight 18 ± 2 g) were purchased from Beijing Huafukang Bioscience Co., Inc. (China) (approval no. SCXK Beijing 2014–0004). All the mice were raised in an environment with the temperature of 23 ± 1 °C with regular 12 h light/dark cycle and allowed free access to feed and water. At the end of the study, all mice were euthanized with carbon dioxide inhalation, and the bilateral thoracotomy was used as second method to confirm the death. In the research, the mice treated in accordance with the Regulations of Beijing Municipality on the administration of laboratory animals, and approved by the Animal Experimental Ethics Committee of the National Institute for Viral Disease Control and Prevention, Chinese Center for Disease Control and Prevention (no. 20180522027).

### Half-life determination

Mice were randomly divided into three groups, with eight in each group. The mice were intraperitoneally injected with 50 μg/kg IFN-1CTPON, IFN-2CTPON, or rhIFN-α2b. Intraocular venous blood was collected at 0.5, 1, 2, 4, 8, and 24 h postinjection. The investigators were blinded to group allocation during the experiments. The IFN concentration was determined with an ELISA kit (Abcam). The data were analyzed with the WinNonLin pharmacokinetic model.

## Data Availability

All the data presented in the article and are available from the corresponding author upon reasonable request.

## Abbreviations

CTP: Carboxyl-terminal peptide CHO Chinese hamster ovary cells TCID Tissue culture infective doses VSV Vesicular stomatitis virus
